# Effects of Supplementing Yeast Fermentation Products on Growth Performance, Colonic Metabolism, and Microbiota of Pigs Challenged with *Salmonella* Typhimurium

**DOI:** 10.3390/ani14243675

**Published:** 2024-12-20

**Authors:** Guoqiang Fan, Yongsen Zhao, Xiaoyi Suo, Yanfei Li, Xiaojing Yang

**Affiliations:** 1 Key Laboratory of Animal Physiology & Biochemistry, Nanjing Agricultural University, Nanjing 210095, China; fan.gq@njau.edu.cn (G.F.); 17826832823@163.com (Y.Z.); sxy@kust.edu.cn (X.S.); 18851173511@163.com (Y.L.); 2 MOE Joint International Research Laboratory of Animal Health and Food Safety, Nanjing Agricultural University, Nanjing 210095, China

**Keywords:** yeast fermentation product, *Salmonella* challenge, colonic inflammation, microbiota, pigs

## Abstract

Yeast fermentation products (YFPs) are known to contain bioactive compounds, such as nutritional metabolites and cell wall polysaccharides (specifically glucan and mannan), which have been demonstrated to exert positive effects on the growth performance and immunity of livestock and poultry. However, the impact of YFPs on intestinal inflammation and microflora composition in pigs infected with *Salmonella* typhimurium remains unclear. To investigate this, a total of 18 weaned pigs were divided into three treatment groups: a non-challenged control group (Con), a group challenged with *Salmonella* typhimurium (ST), and a group challenged with *Salmonella* typhimurium and supplemented with 0.4% YFP (YFP). The experiment spanned five weeks, encompassing a period of 21 days prior to and 14 days subsequent to the initial *Salmonella* typhimurium challenge. The findings indicated that the YFP group exhibited an increase in average daily gain (ADG) and a decrease in the feed-gain ratio (F/G) in comparison to the ST group following the *Salmonella* challenge. Additionally, the YFP group demonstrated a reduction in the levels of inflammatory cytokines in plasma and a decrease in the expression of inflammatory genes in the colon. Treatment with YFP also resulted in improved colon histomorphology, heightened alpha diversity of the gut microbiota, augmented the abundance of butyrate-producing bacteria, and elevated concentrations of short-chain fatty acids (SCFAs). In addition, YFP reprogrammed energy metabolism in colon epithelial cells by blunting glycolysis. Together, dietary YFP supplementation alleviated colon inflammation in weaned pigs challenged with *Salmonella* typhimurium, and shaped the beneficial microbiota, thereby maintaining gut homeostasis. The results provided evidence supporting the application of yeast fermentation products in livestock production.

## Simple Summary

The pig industry is increasingly seeking natural, safe, and cost-effective feed additives due to the prohibition of antibiotics and growing production demands. Yeast fermentation products have demonstrated antioxidant and anti-inflammatory properties in feed additive studies. However, the mechanism by which yeast fermentation product supplementation improves the performance and health status of weaned piglets needs to be more thoroughly investigated. The results of this study showed that yeast fermentation product supplementation alleviated colon inflammation in weaned piglets challenged with *Salmonella* typhimurium, and shaped the beneficial microbiota, thereby maintaining gut homeostasis. These findings provide a basis for considering yeast fermentation products as valuable feed additives in the pig industry.

## 1. Introduction

*Salmonella* enterica serovar typhimurium is a pathogenic bacterium of clinical significance, that causes food-borne zoonotic infections worldwide [1]. Pigs serve as an important infection reservoir for humans due to their ability to carry a broad range of *Salmonella* serovars [2]. *Salmonella* infection diminishes gut microbiota diversity, causing poor growth performance [3]. The administration of antibiotics is the primary treatment for *Salmonella*-related disease, but it also contributes to the spread of drug resistance, a significant global public health issue. Notably, antibiotic treatments increase the risk of *Salmonella* developing resistance over time [4].

The intestine is regarded as the ecological niche of *Salmonella*, with the intestinal mucosa performing a crucial role in regulating the immune response to bacterial infection [5]. In the past few years, there has been increasing interest in the role of gut microbiota in the susceptibility to intestinal pathogens. The microbiota contributes to the development and maintenance of the mucosal immune system and acts as a barrier against bacteria invading the epithelial layer, where it competes for nutrients with the host microbiota [6,7]. It has been shown that *Salmonella* can disrupt the composition of the gut microbiota through its virulence factors, leading to an inflammatory mucosal response [8]. Moreover, evidence also shows that *Salmonella* can alter the metabolic pattern of colon epithelial cells via its virulence factors [9]. Thus, mitigating the influence of *Salmonella* digestive tract infections on animals remains a major concern for the livestock industry.

Yeast fermentation products (YFPs) contain bioactive compounds, including nucleotides, nutritional metabolites, and cell wall polysaccharides (specifically β-glucan and mannan). Previous studies have investigated the impact of yeast cell wall components on the immune function of weaning pigs [10,11,12]. It has been reported that yeast β-glucans alleviate the elevation of pro-inflammatory cytokine levels in LPS or *Escherichia* coli-challenged pigs [13,14]. Additionally, dietary supplementation with yeast-derived mannans could improve weight gain and enhance the percentage of immune cells in pigs [15]. However, the effects of yeast fermentation products on digestive tract inflammation and microbiota composition in pigs challenged with *Salmonella* typhimurium have not been reported.

Therefore, this study employed weaned pigs challenged with *Salmonella* typhimurium and supplemented with yeast fermentation products. The results indicate how yeast fermentation products influence colon epithelial cell metabolism and microbiota composition of pigs challenged with *Salmonella* typhimurium. Our study provides a potentially effective way to prevent the adverse effects of *Salmonella* infection in livestock production.

## 2. Materials and Methods

### 2.1. Ethics Approval

All procedures involving animals were approved by the Institutional Animal Care and Use Committee (IACUC) of Nanjing Agricultural University (IACUC approval number: PT2019015). The sampling procedures followed the “Guidelines on Ethical Treatment of Experimental Animals” (2006) No. 398, set by the Ministry of Science and Technology, China. In this study, all experimental methods were performed in accordance with the Nanjing Agricultural University Health Guide for the Care and Use of Laboratory Animals.

### 2.2. Animals and Experimental Design

A total of 18 weaned male pigs (Duroc × Yorkshire × Landrace) were housed at the Laboratory Animal Center of Nanjing Agricultural University. The pigs used did not receive *Salmonella* typhimurium vaccines, antibiotic injections, or antibiotics in feed. All pigs used in this study were susceptible to *Salmonella* typhimurium. They were allowed free access to feed and water. The pigs were housed in pens (pen size: 1.2 m × 1 m) in an environmentally controlled nursery building. The pens were equipped with a self-feeder, a nipple drinker, and plastic-covered expanded metal floors in an environmentally controlled building (temperature maintained at 25–28 °C with a 16 h light and 8 h dark cycle).

All pigs were randomly allotted to three dietary treatments (n = 6) based on the initial body weight: (1) negative control (Con): control diet, without *Salmonella* typhimurium challenge; (2) positive control (ST): control diet, with *Salmonella* typhimurium challenge; (3) YFP: control diet plus 4 g YFP/kg feed with *Salmonella* typhimurium challenge. The yeast fermentation products were derived from the fermentation of Saccharomyces cerevisiae and were provided by the Cargill Company (XPC, Diamond V, Cedar Rapids, IA, USA). The main components of YFPs include mannan-oligosaccharides, beta-glucan, proteins, peptides, amino acids, organic acids, vitamins, mineral salts, and nucleotides. After 21 days on their experimental diets, the pigs in the ST and YFP groups were orally inoculated with a 5 mL suspension containing 10^9^ CFU of *Salmonella* typhimurium. A week later, the second challenge was performed the same as the first. The body weight of each piglet was recorded on days 0, 7, 14, 21, and 35, and the feed consumption per pen was recorded every day of the experiment to calculate the average daily gain (ADG), average daily feed intake (ADFI), and feed efficiency (F/G) from day 1 to 21 (pre-challenge) and day 21 to 35 (post-challenge). The experimental diet is described in detail in Table 1.

### 2.3. Sample Collections

All pigs were weighed and then sacrificed via exsanguination at the end of the study. Blood samples were collected into heparinized tubes, and plasma was separated through centrifugation at 3000× *g* for 15 min and stored at −80 °C until analysis. Fresh colon content was collected and immediately frozen in liquid nitrogen to isolate bacterial genomic DNA and analyze SCFAs. Colon mucosa was obtained as described previously [16], immediately frozen in liquid nitrogen, and stored at −80 °C for further analysis.

### 2.4. Biochemical Analysis

The concentrations of interleukin (IL), including IL-1β, IL-6, IL-18, and tumor necrosis factor-α (TNFα) in plasma and IL-1β, IL-6, and TNFα in the colonic mucosa, were measured using porcine-specific ELISA kits (Jiangsu Meimian Industrial Co., Ltd., Yancheng, China). Total protein (TP), globulins (GLOB), albumin (ALB), and lactate dehydrogenase (LDH) were measured with a biochemical automatic analyzer (Hitachi 7020, HITACHI, Tokyo, Japan) using commercial assay kits (Wako Pure Chemical Industries, Ltd., Wako, Japan).

### 2.5. Histomorphology Analysis

The paraformaldehyde-fixed colon was dehydrated in graded alcohol and embedded in paraffin wax. Then, hematoxylin and eosin (H&E)-stained paraffin sections were viewed under a bright field using the Pannoramic SCAN II, and images were captured with 3DHISTECH software V1.0 (3DHISTECH Ltd., Budapest, Hungary). The degree of intestinal tissue damage was scored as described previously [17,18], evaluating the extent of epithelial loss on intestinal villi and inflammatory infiltration, which were included in the histopathological examination.

### 2.6. Colon Microbiota Analysis

The extraction of total genomic DNA from the colonic content was performed following the standard protocol of the QIAamp DNA stool Mini Kit (QIAGEN, Hilden, Germany). The integrity of DNA isolation was measured using 1% agarose gel electrophoresis. The V3–V4 region of the 16S rRNA gene was amplified using a specific primer. Amplicon library sequencing was performed on an Illumine Hiseq 2500 platform (Illumina, San Diego, CA, USA) using standard protocols. All raw sequence reads involved in our study were deposited in the NCBI Sequence read archive (SRA). The alpha diversity (Ace, Chao1, Shannon, and Simpson) was measured using the MOTHUR program (version v.1.30; http://www.mothur.org).

### 2.7. Metabolite Analysis

Gas chromatography was used to determine the concentrations of SCFAs in the colonic content according to the method outlined in Reference [19]. Briefly, 0.30 g of each sample was added to sterile tubes containing 1.5 mL of distilled water. Each mixture was shaken strongly for 5 min and then centrifuged at 12,000× *g* for 10 min at 4 °C and filtered with 0.22 μm mesh. Finally, 1 μL of supernatant was injected into the Agilent 7890B Gas Chromatograph (Agilent Technologies, Santa Clara, CA, USA). The concentrations of lactate in colon mucosa were measured using an assay kit, following the protocol provided by Nanjing Jiancheng Biological Engineering Institute, Nanjing, China.

### 2.8. Total RNA Isolation and Real-Time Quantitative PCR

Total RNA was isolated from 30 mg of frozen colonic mucosa with 1 mL of TRIzol (Sango Biotech, Shanghai, China) and reverse-transcribed according to the manufacturer’s protocol (Vazyme Biotech, Nanjing, China). Diluted cDNA (2 μL, 1:25) was used as a template for real-time PCR, which was performed on a real-time PCR system (Mx3000P, Agilent Technologies, Santa Clara, CA, USA). Moreover, β-actin was chosen as a reference gene to normalize the mRNA abundance of target genes. The primer sequences of the target genes are listed in Table 2. The 2^−ΔΔCT^ method was used to analyze real-time PCR data.

### 2.9. Western Blot Analysis

Total protein was extracted from 50 mg of frozen colonic mucosa as previously described [20]. The protein concentration was measured using a Pierce BCA Protein Assay kit (No. 23225, Thermo Scientific, Waltham, MA, USA) according to the manufacturer’s instructions. Western blot analysis of Occludin (DF7504, Affinity, Liyang, China, diluted 1:1000) was carried out. β-actin (AC026, ABclonal, Wuhan, China, diluted 1:50,000) was used as the internal control.

### 2.10. Statistical Analysis

All data were checked for normality using exploratory analysis. Data were analyzed using SPSS 21.0 for Windows (SPSS Inc., Chicago, IL, USA). All data were analyzed using one-way ANOVA, with the YFP diet as a fixed factor, and Duncan’s test was used to determine the difference among groups. The pigs were recognized as a statistical unit. The values were presented as the means ± SEM with significance at *p* < 0.05, and 0.05 < *p* < 0.10 considered a tendency. The correlation between differential gut microbiota and plasma parameters or SCFAs and gene expression was analyzed using Pearson correlation analysis with the Pheatmap package in R (version 4.1.2).

## 3. Results

### 3.1. Growth Performance

*Salmonella* typhimurium administration tended (*p* = 0.09) to decrease the average daily gain (ADG) of pigs compared with the Con group, yet YFP increased (*p* < 0.05) the ADG of pigs compared with the ST group. The feed/gain ratio (F/G) was observed to significantly increase (*p* < 0.05) in *Salmonella* typhimurium-challenged piglets compared with those in the Con group, a finding that was reversed by the YFP (*p* < 0.05). No significant changes were observed in ADG or F/G among the three groups before *Salmonella* typhimurium treatment (Table 3).

### 3.2. Biochemical Index Analysis of Plasma

**Figure 1:**
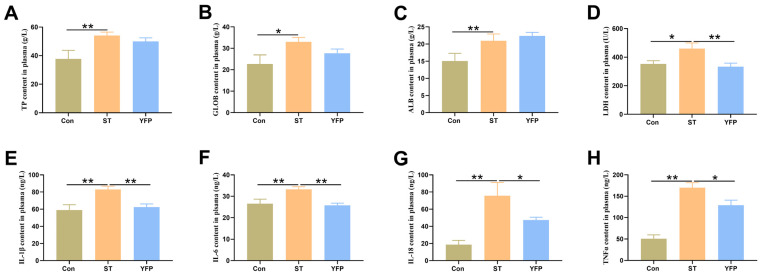
The effects of YFPs on the concentrations of plasma biochemical index and the contents of inflammatory cytokines in plasma of pigs. (**A**) TP content in plasma; (**B**) GLOB content in plasma; (**C**) ALB content in plasma; (**D**) LDH content in plasma; (**E**) IL-Iβ content in plasma; (**F**) IL-6 content in plasma; (**G**) IL-I8 content in plasma; (**H**) TNFα content in plasma. The values are presented as mean ± SEM, n = 6. * *p* < 0.05, ** *p* < 0.01.

*Salmonella* typhimurium challenge markedly increased (*p* < 0.05) the concentrations of total protein (TP), globulin (GLOB), albumin (ALB), and lactic dehydrogenase (LDH) (Figure 1A–D) in plasma compared with the Con group. Dietary YFP supplementation did not protect the pigs from these changes except LDH. Additionally, the concentrations of inflammatory cytokines interleukin (IL-1β, IL-6, and IL-18) (Figure 1E–G) and TNFα (Figure 1H) in plasma were higher (*p* < 0.05) in the ST pigs compared to the Con pigs. Compared with ST pigs, the concentrations of IL-1β, IL-6, IL-18, and TNFα were lower (*p* < 0.05) in YFP pigs (Figure 1E,H).

### 3.3. Colon Morphology

**Figure 2:**
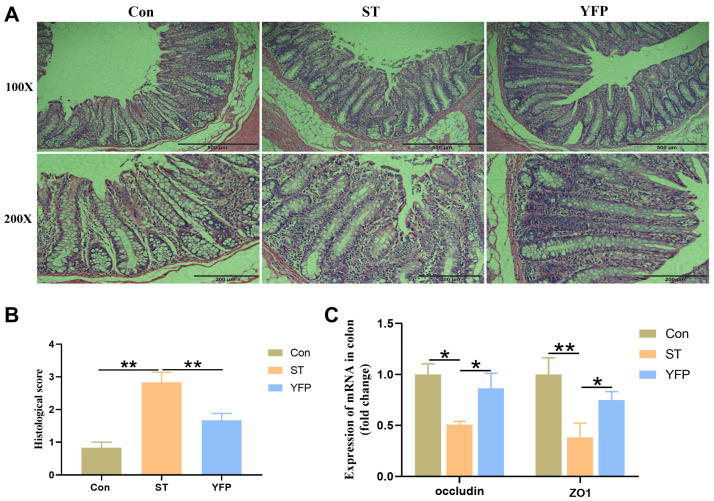
The effects of YFPs on colon injury. (**A**) Morphology of the colon; (**B**) Histological score of colon tissue; (**C**) The mRNA expression of Occludin and ZO1 in the colon. The values are presented as mean ± SEM, n = 6. * *p* < 0.05, ** *p* < 0.01.

*Salmonella* typhimurium-infected pigs showed increased (*p* < 0.05) infiltration of inflammatory cells and higher histological scores in the colon mucosa compared to the Con pigs, which could be reversed by dietary YFP supplementation (Figure 2A,B). The ST group showed reduced (*p* < 0.05) Occludin and tight junction ZO1 expression levels in the colon compared with the Con group. In contrast, pigs fed with a YFP had higher (*p* < 0.05) ZO1 and Occludin expression compared with the ST group (Figure 2C).

### 3.4. Genes Expression and Metabolite Analysis of Colonic Mucosa Related to Glycolysis, and Colonic Inflammation Cytokine Expression

**Figure 3:**
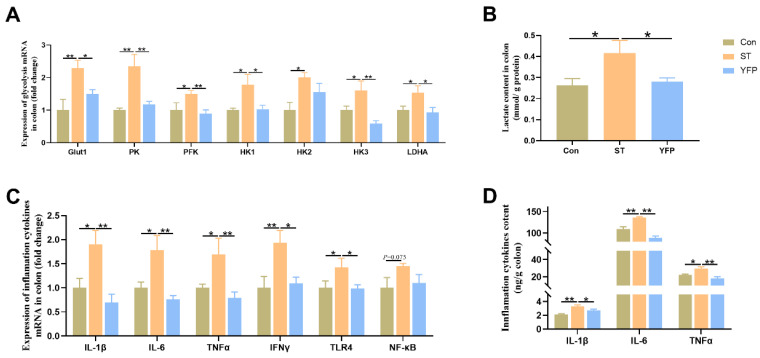
The effects of YFP on glycolysis and inflammation levels. (**A**) Glycolysis-related mRNA expression; (**B**) The concentration of lactate in the colon; (**C**) Inflammatory cytokines mRNA expression; (**D**) Inflammatory cytokine content in the colon. The values are presented as mean ± SEM, n = 6. * *p* < 0.05, ** *p* < 0.01.

To examine whether YFP influences glycolysis, we measured glycolysis-related genes and lactate levels in the colonic mucosa. *Salmonella* typhimurium challenge significantly increased (*p* < 0.05) the glycolysis-related mRNA expression of glucose transporter 1 (Glut1), pyruvate kinase (PK), phosphofructokinase (PFK), hexokinase1 (HK1), hexokinase3 (HK3), and lactate dehydrogenase A (LDHA) (Figure 3A); meanwhile, the concentrations of lactate in the colon mucosa were higher (*p* < 0.05) compared to those in the Con group (Figure 3B). However, dietary YFP supplementation decreased (*p* < 0.05) the expression of these genes as well as the concentration of lactate in the colonic mucosa (Figure 3A,B). From these results, it could be deduced that YFPs could alleviate colonic mucosa metabolic disorders induced by *Salmonella* typhimurium.

*Salmonella* typhimurium challenge significantly increased (*p* < 0.05) the mRNA expression of IL-1β, IL-6, TNFα, IFNγ, and TLR4 (Figure 3C) and the concentrations of IL-1β, IL-6, and TNFα in the colonic mucosa (Figure 3D). However, YFP supplementation could decrease the mRNA expression and the concentrations of inflammatory cytokines.

### 3.5. Metabolite Analysis of Colonic Content

**Figure 4:**
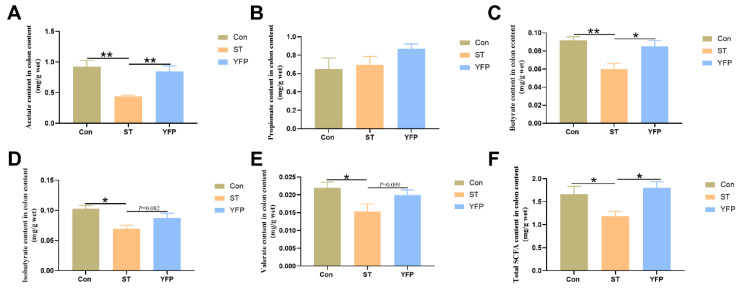
The effects of YFP on concentrations of SCFAs in colon content. (**A**) Acetate content in colon content; (**B**) Propionate content in colon content; (**C**) Butyrate content in colon content; (**D**) Isobutyrate content in colon content; (**E**) Valerate content in colon content; (**F**) Total SCFA content in colon content. The values are presented as mean ± SEM, n = 6. * *p* < 0.05, ** *p* < 0.01.

To evaluate whether alterations to the gut microbial composition influenced the fermentative capacity, the concentrations of colonic-content SCFAs were determined. *Salmonella* typhimurium challenge significantly decreased (*p* < 0.05) the concentrations of acetate, butyrate, isobutyrate, valerate, and total SCFA compared to the Con group. Dietary YFP supplementation significantly increased (*p* < 0.05) the concentrations of acetate, butyrate, and total SCFA. However, the *Salmonella* typhimurium challenge had no influence on the propionate content in the colon content (Figure 4A–F).

### 3.6. Microbiota Composition of Colonic Content

**Figure 5:**
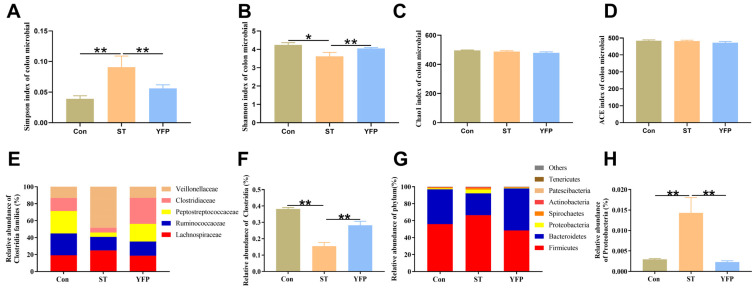
The effects of YFP on the microbial content of the colon. (**A**) Simpson index of microbial in colon content; (**B**) Shannon index of microbial in colon content; (**C**) Chao1 index of microbial in colon content; (**D**) ACE index of microbial in colon content; (**E**) Families of butyrate-producer content of colon content; (**F**) Clostridia content of colon content; (**G**) Abundant phyla of colon content; (**H**) Proteobacteria content of colon content. The values are presented as mean ± SEM, n = 4. * *p* < 0.05, ** *p* < 0.01.

The effects of dietary YFPs on the gut microbiota of pigs under the *Salmonella* typhimurium challenge have rarely been reported. To confirm this issue, we analyzed colonic microbiota compositions. Alpha diversity analysis showed a significant difference (*p* < 0.05) in community diversity based on the Simpson and Shannon indices (Figure 5A,B); however, no significant differences were observed in Chao1 and ACE community richness (Figure 5C,D).

We investigated whether dietary YFP supplementation would influence butyrate-producing bacteria. ST treatment significantly reduced the abundance of *Clostridia* (phylum Firmicutes) (0.16%) compared to the Con group (0.38%), which are *obligate anaerobes* that include abundant butyrate-producers, specifically Ruminococcaceae (25.73% in Con, 16.01% in ST and 16.63% in YFP), Peptostreptococcaceae (26.50% in Con, 5.07% in ST and 20.88% in YFP) and Clostridiaceae (15.36% in Con, 5.70% in ST and 30.67% in YFP) (Figure 5E,F).

We found that the predominant phyla in the colon content at the phylum level were Firmicutes (55.79% in Con, 66.50% in ST, and 48.48% in YFP), Bacteroidetes (41.08% in Con, 25.75% in ST, and 49.51% in YFP), and Proteobacteria (1.18% in Con, 4.29% in ST, and 0.92% in YFP) were. The relative abundance of Proteobacteria, a common marker of gut dysbiosis, was higher in the ST group (4.29%) as compared to the Con group (1.18%). YFP supplementation prevented the expansion of Proteobacteria (0.92%) (Figure 5G,H).

### 3.7. Correlation Analysis of the Gut Microbiota, Plasma Parameters, and SCFAs of the Colon Content

**Figure 6:**
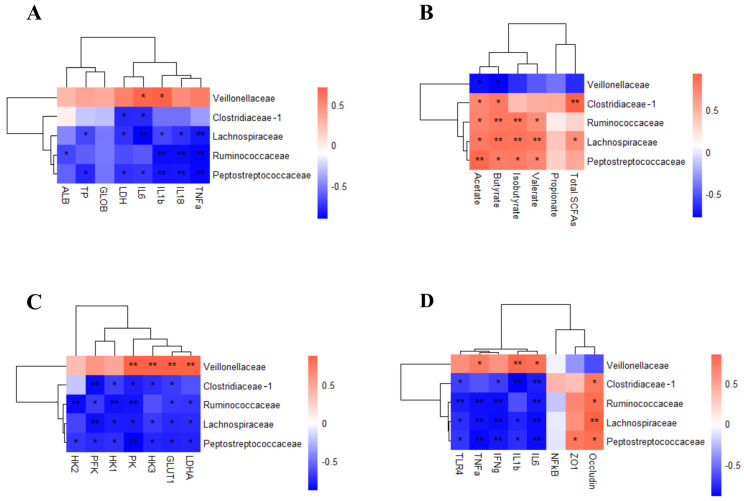
Pearson correlation analysis between the colonic microbiota and metabolism and inflammation. (**A**) Relationship between colonic microbiota and inflammation markers; (**B**) Relationship between colonic microbiota and organic acids; (**C**) Relationship between colonic microbiota and glycolysis gene expression; (**D**) Relationship between colonic microbiota and colon inflammatory cytokines. n = 4. * *p* < 0.05, ** *p* < 0.01.

To understand how the differential gut microbiota community of predominant families impacts host metabolism, a Pearson correlation matrix was generated to explore these relationships. We detected the correlations between colon differential microbiota related to SCFAs, glycolysis, and inflammatory cytokines. The relative abundance of Peptostreptococcaceae, Ruminococcaceae, and Lachnospiraceae showed a negative correlation with IL-1β, IL-18, and TNFα levels in plasma. The relative abundance of Lachnospiraceae and Clostridiaceae_1 showed a negative correlation with IL-6 and LDH levels in plasma. The relative abundance of Veillonellaceae showed a positive correlation with IL-1β and IL-6 levels in plasma (Figure 6A) (*p* < 0.05).

Peptostreptococcaceae, Lachnospiraceae, and Ruminococcaceae were positively correlated with acetate, butyrate, isobutyrate, and valerate. Clostridiaceae_1 was positively correlated with acetate, butyrate, and total SCFAs. Veillonellaceae was negatively correlated with acetate and butyrate (Figure 6B) (*p* < 0.05).

Peptostreptococcaceae, Ruminococcaceae, Lachnospiraceae, and Clostridiaceae_1 were negatively correlated with glycolysis-related genes such as PK, PFK, HK1, HK3, and Glut1. The gene expression of PK, HK3, Glut1, and LDHA was positively correlated with Veillonellaceae (Figure 6C) (*p* < 0.05).

To determine whether the gut microbiota influences intestinal barrier function, the correlations between intestinal barrier gene expression levels and differential microbiota families were measured. The relative abundance of Peptostreptococcaceae, Lachnospiraceae, and Ruminococcaceae were negatively correlated with IL-6, IFNγ, TNFα, and TLR4. Clostridiaceae_1 was negatively correlated with IL-6, IL-1β, IFNγ, and TLR4. Peptostreptococcaceae and Lachnospiraceae were negatively correlated with IL-1β. The relative abundance of Veillonellaceae was positively correlated with IL-6, IL-1β, and TNFα. The gene expression of tight junction protein Occludin was positively correlated with Peptostreptococcaceae, Lachnospiraceae, Ruminococcaceae, and Clostridiaceae_1. Similarly, the gene expression of tight junction protein ZO1 was positively correlated with Peptostreptococcaceae (Figure 6D) (*p* < 0.05).

## 4. Discussion

Yeast fermentation products have been used in the livestock industry, but the mechanism of action is largely unclear, especially in inflammation in animals. In this study, during the pre-challenge period, supplementation with YFPs had no significant effect on the growth performance of pigs compared with the control group. However, during the *Salmonella* typhimurium challenge period, YFPs significantly enhanced ADG. Moreover, YFPs decreased the colon inflammation levels and improved the intestinal microbial community compared to the ST group. The present study indicates that YFPs provide colon protection against *Salmonella* typhimurium.

It has been shown that yeast supplementation increases body weight and ADG, and yeast supplementation decreases the total bacteria and lactobacilli in the feces of nursery pigs [21]. Sows fed with *Bacillus* spp. and yeast extracts derived from *S. cerevisiae* increased ADG in piglets, but pigs fed yeast tended to demonstrate decreased ADG in the nursery. Sows fed with live yeast (*S. cerevisiae* strain *NCYC Sc 47*) and yeast-based prebiotics derived from S. cerevisiae improved pigs’ growth in the nursery period [22]. These studies show that feeding yeast at different growth stages of pigs had different effects on growth performance. In this study, weaned pigs fed with YFPs showed a significant increase in growth performance after a *Salmonella* typhimurium challenge.

The enteric pathogen *Salmonella* can overcome niche protection through its virulence factors to induce intestinal inflammation by stimulating the production of cytokines such as IL-1, IL-6, and TNFα [23]. In the present study, increased IL-1β, IL-6, IL-18, and TNFα concentrations in plasma and IL-1β, IL-6, and TNFα concentrations in the colon indicated that the intestine successfully induced inflammation after *Salmonella* treatment. Pigs receiving YFPs had lower concentrations of IL-1β, IL-6, IL-18, and TNFα in plasma and IL-1β, IL-6, and TNFα in the colonic mucosa compared to the ST group, implying that YFPs decreased the inflammation reaction.

Intestinal morphology can be used as an indicator of intestinal health. In response to inflammation induced by pathogens, deeper crypts exhibit faster cellular turnover, allowing the renewal of villi as needed [24]. In this current study, dietary YFP supplementation improved intestinal histomorphology in infected pigs. Tight junctions between intestinal epithelial cells, which protect the body from intestinal pathogens, influence intestinal mucosal barrier function to a great extent [25]. In this current study, YFP supplementation significantly increased tight junction ZO1 and Occludin expression in the colon, which is expected to improve intestinal mucosal barrier function. Our results demonstrated that YFPs maintain intestinal integrity and barrier function, partly by elevating intestinal tight junction protein expression.

There are a large number of microbial communities in the gastrointestinal tracts of humans and animals, which play a crucial role in the development of intestinal function and the host’s metabolism and immune system [26]. According to our current study, YFPs significantly improved the decreased microbiota diversity and altered the microbiota structure of the colonic microbiota communities. There is increasing interest in the crosstalk between dietary supplementation and gut microbiota to improve the production of SCFAs, mainly from Bacteroidetes and Firmicutes phyla in the colon [27]. SCFAs in the colon, including dominant acetate, propionate, and butyrate, are an energy source for epithelial cells and play an important role in inflammatory and immune signaling [28]. Our study found that YFP could decrease the ratio of Firmicutes and Bacteroidetes at the phylum level, which is related to the metabolism level. Meanwhile, dietary YFPs increased the number of butyrate-producing bacteria, such as Ruminococcaceae, Peptostreptococcaceae, and Clostridiaceae, at the family level, thereby promoting the production of acetate and butyrate. Additionally, butyrate contributes to the anti-inflammation reaction, as the relative abundance of butyrate-produced bacteria was negatively correlated with the production of inflammatory cytokines.

There is an increasing amount of evidence showing that some bacterial pathogens can alter the pattern of energy metabolism to obtain nutrients from host cells and support their own growth and long-term persistence in host tissue [29,30,31]. However, these studies were limited to mice and were not replicated in pigs. In addition, the effect of YFPs on intestinal energy metabolism in *Salmonella*-infected pigs has not been reported. Therefore, we detected the gene expression and metabolites related to glycolysis in colon epithelial cells. We found that *Salmonella* typhimurium increased the expression of glycolysis-related genes, including PK, PFK, HK1, HK2, HK3, Glut1, and LDHA, and the concentration of lactate in colon epithelial cells. Dietary YFP supplementation reprogrammed the energy metabolism of colon epithelial cells by blunting the expression of PK, PFK, HK1, HK3, Glut1, and LDHA, and the concentration of lactate. The results showed that Veillonellaceae promoted colonic inflammation and anaerobic glycolysis of colonic epithelial cells and had a destructive effect on the colonic mucosal barrier. The short-chain fatty acid-producing bacteria Ruminococcaceae, Peptostreptococcaceae, Lachnospiraceae, and Clostridiaceae increase the concentration of short-chain fatty acids in the colon, inhibit colon inflammation, and inhibit anaerobic glycolysis of colon epithelial cells.

## 5. Conclusions

Collectively, dietary YFP supplementation positively influenced growth performance and intestinal barrier function while reducing intestinal inflammation in weaned pigs challenged with *Salmonella* typhimurium. Additionally, YFPs could reprogram the energy metabolism of colon epithelial cells and shape the beneficial microbiota, thereby maintaining gut homeostasis.

## Figures and Tables

**Table 1:** Ingredient composition of experimental diets (%, as-fed basis).

Ingredient	Percentage (%)
Corn	60.00
Soybean meal	30.00
Fish meal	6.60
Lys	0.12
CaHPO_3_	0.80
Rock powder	0.88
Salt	0.60
1% Premix ^a^	1.00
Calculated nutrient content	
DE, kcal/kg	3283
CP, %	18
Ca, %	0.80
Total P, %	0.60

Rock powder: Limestone, the main ingredient is CaCO_3,_ providing calcium. ^a^ Premix provided the following amounts of vitamins and minerals per kilogram of feed on an as-fed basis: vitamin A: 11,000–13,000 IU, vitamin D3: 3000–4000 IU, vitamin E: 3000–4000 IU, vitamin K3: 6 mg, vitamin B2: 10 mg, vitamin B6: 5 mg, vitamin B12: 0.5 mg, niacin: 70 mg, pantothenic acid: 550 mg, folic acid: 40 mg, biotin: 5 mg, choline: 30 mg, Mn: 40 mg, Fe: 200 mg, Zn: 300 mg, I: 2 mg, Cu: 350 mg, and Se: 10 mg.

**Table 2:** The primer sequences of the target genes for RT-PCR.

Target Genes	Primer Sequences (5′ to 3′)	
IL-1β	F: ACATGCTGAAGGCTCTCCAC	R: CAGGGTGGGCGTGTTATCTT
IL-6	F: GCAGTCACAGAACGAGTGGA	R: CTCAGGCTGAACTGCAGGAA
IFNγ	F: AGCTTTTCAGCTTTGCGTGA	R: TGCTCCTTTGAATGGCCTGG
TNFα	F: GCCCTTCCACCAACGTTTTC	R: CAAGGGCTCTTGATGGCAGA
TLR4	F: CGTGCAGGTGGTTCCTAACA	R: AAAGGCTCCCAGGGCTAAAC
NFκB	F: CGGGGACTACGACCTGAATG	R: CTTTCTGCACCTTGTCGCAC
ZO1	F: CTGCCAAGTGAAACTGCACA	R: TATCAAACTCAGGAGGCGGC
Occludin	F: CCTCCTCCCCTTTCGGACTA	R: TCACTTTCCCGTTGGACGAG
PK	F: ATTATTTGAGGAACTCCGCCGCCT	R: ATTCCGGGTCACAGCAATGATGG
PFK	F: AGGGCCTTGTCATCATTGGG	R: ACTGCTTCCTGCCTTCCATC
HK1	F: CGCGCAACTACTGGCATATT	R: TTCATCAGAGAGCCGCATGG
HK2	F: ATCGCCTGCTTATTCACGGA	R: TCGCCATGTTCTGTCCCATC
HK3	F: ATCCTGGAAGATCTGGGGCT	R: CGACACCAGCTTGGAGAAGT
Glut1	F: ATGGATCCCAGCAGCAAGAAG	R: AGCGGTGGTTCCATGTTTGA
LDHA	F: CAAGGAGCAGTGGAAGGAGG	R: CCAAGTCTGCCACAGAGAGG

**Table 3:** The effects of YFPs on growth performance.

Items	Con	ST	YFP
Pre-challenge			
ADG (Kg)	0.49 ± 0.02	0.45 ± 0.03	0.48 ± 0.02
ADFI (Kg)	0.73 ± 0.02	0.80 ± 0.02	0.76 ± 0.03
F/G	1.51 ± 0.07	1.72 ± 0.09	1.68 ± 0.06
Post-challenge			
ADG (Kg)	0.66 ± 0.03	0.51 ± 0.04	0.69 ± 0.04 ^#^
ADFI (Kg)	1.13 ± 0.02	1.05 ± 0.06	1.15 ± 0.04
F/G	1.76 ± 0.09	2.09 ± 0.03 *	1.81 ± 0.09 ^#^

The values are presented as mean ± SEM. * *p* < 0.05, # *p* < 0.05. *: Compared with the Con group, #: Compared with the ST group; ADG: average daily gain; ADFI: average daily food intake.

## Data Availability

Original data are available from the corresponding author upon reasonable request.
